# Supplementary material for: Differences in codon usage between host-species-specific rabies virus clades are driven by non-GC3 nucleotide composition
Source: Virus Evol. 2026 Jul 1;12(1):veag039. doi: 10.1093/ve/veag039 (PMC13403185; doi:10.1093/ve/veag039)
Supplement: Supplementary_materials_veag039 [file supplementary_materials_veag039.docx]

## Supplementary material

**Supplementary Table S1: Accession numbers for the sequences used in our analysis by host species.**

| **Clade (Host)** | **Accession numbers** |
| --- | --- |
| Asian SEA2b (Chinese ferret badger) | FJ598135, FJ712195, FJ712196, FJ719751, FJ719753, FJ719755, GU647092, HQ118114, JN974877, JQ950446, JQ950448, KF726852, KP319185, KP319186, KP319187, KP319188, KP319191, KP319193, KP319194, KP319195, KP319199, KP319201, KP319203, KP319204, KP319205, KP319206, KP319207, MT068612, MT068615 |
| Asian SEA2a (dog) | DQ666289, DQ666290, DQ866105, DQ866106, DQ866108, DQ866109, DQ866110, DQ866112, DQ866116, DQ866119, EU086182, EU086183, EU828653, FJ561726, FJ561727, FJ561728, FJ866829, FJ866830, FJ866831, GQ472468, GQ472470, GQ472473, GQ472477, GU358653, HM486355, HM486356, HM486368, HM486369, HM486374, HM486379, HM486381, JF819605, JF819615, JN974823, JN974824, JN974828, JN974829, JN974838, JN974842, JN974845, JN974870, JN974871, JN974872, JN974873, JQ970480, KT221107, KT894558, KT894572, KT894573, KT894577, KT894578, KX148264, KY451767, MG201879, MG201880, MG201884, MG201919, MG201921, MN784132 |
| Arctic A (Arctic fox) | AY352488, AY352501, JN258588, JN258592, JX987747, KU198460, KU198462, KU198464, KU198466, KU198467, KU198472, KU198476, KU198477, KU198478, KX036361, KX036362, KX036364, KX036365, KX036366, KX954123, LT598537, LT598538, LT598539, LT598540, LT598541, LT598543, LT909550, MG458313, MN233885, MN233886, MN233892, MN233896, MN233900, MN233948, MN233949, MN233950, MN233952, MN233953, MN233955, MN233956, MN233958, MN233960, MN233963, MN233967, MN233968, MN233969, MN233973, MN233974, MN233975, MN233977, MN233978, MN233982, MN233985, MN233988, MN233991, MN233992, MN233998, MN234000, MN234002, MN234004, MN234008, MN234016, MN234017, MN234018, MN234023, MN234043, MN234046, MN234055, MN384717 |
| Cosmopolitan AF1b (dog) | AB284509, AB284511, AB284512, AB284513, AB284514, EU853582, EU853584, EU853586, EU853587, EU853590, HM179504, HM179505, HM179506, KF155002, KR534217, KR534218, KR534219, KR534228, KR534229, KR534230, KR534233, KR534238, KR534244, KR534250, KR534251, KR534252, KR534253, KR906735, KR906736, KR906739, KR906741, KR906742, KR906744, KR906745, KR906746, KR906747, KR906748, KR906749, KR906751, KR906756, KR906757, KR906759, KR906762, KR906763, KR906765, KR906766, KR906767, KR906770, KR906773, KR906780, KR906784, KR906790, KR906792, KT336432, KT336433, KT336434, KT336435, KT336436, KT336437, KX148203, KX148204, KX148206, KX148208, KY210224, KY210243, KY210247, KY210249, KY210253, KY210263, KY210264, KY210279, KY210287, KY210304, KY563717, LC029890, MN726804, MN726826, MN726827, MT454631, MT454635, MT454637, MT454641, MT454643, MT454644, MT454645, OK317992, OK317993, OK317995, OK317996, OK317998, OK317999, OP375406, OP375407, OP375410, OP375413, OP375414, OP375415, OP375418, OP375421, OP375423, OR045939, OR045949, OR270973, OR270979, OR270997, OR270998, OR270999, OR271001, OR271003, PV855388, PV855390, PV855392, PV855393, PV855394, PV855397, PV855415 |
| Bat LC (hoary bat) | AF351845, AF351846, AF351858, AF394883, AF394884, GU644712, GU644713, GU644714, GU644715, GU644716, GU644717, GU644718, GU644719, GU644720, GU644721, JQ685947 |
| Cosmopolitan AM2a (mongoose) | AY854505, AY854525, AY854531, AY854544, AY854546, AY854558, AY854567, AY854573, AY854576 |
| RAC-SK SCSK (skunk) | EU345002, EU345003, EU345004, JQ685938, JQ685968, MW055084, MW055085, MW055086, MW055087 |
| Bat DR (vampire bat) | AB519642, AF070449, AF351847, AF351852, AY854587, AY854592, AY854594, AY877433, AY877434, AY877435, GU592648, KF656696, KF864234, KF864322, KF864397, KM594040, KM594041, KM594042, KP202393, KT023101, MN968374, MN968375, MN968377, MN968386, MT891038, MW249020, MW579786, MW579820, MW579824, MW579833, PQ379829, PQ379831, PQ379832, PQ379834, PQ379836, PQ379849, PQ510120, PQ603086, PQ671599, PQ671601 |
| Bat EF-E2 (big brown bat) | AF351828, AF351831, AF351832, AF351833, AF351855, AF351861, AF351862, AY039227, AY039228, AY039229, GU644652, GU644654, GU644655, GU644656, GU644657, GU644658, GU644659, GU644660, GU644661, GU644662, GU644663, GU644664, GU644665, GU644666, GU644667, GU644668, GU644669, GU644670, GU644671, GU644676, GU644677, GU644684, GU644689, GU644690, GU644695 |
| Bat TB1 (Mexican free-tailed bat) | AF351849, AF394876, GU644760, GU644761, GU644762, GU644763, GU644764, GU644765, GU644766, GU644767, GU644768, GU644769, GU644770, GU644771, GU644772, GU644773, GU644774, GU644775, GU644776, GU644777, GU644778, GU644779, GU644780, GU644781, GU644782, GU644784, GU644785, GU644786, GU644787, GU644788, JQ685905 |

**Supplementary Table S2: Mean RSCU values across different clades.** Green highlight indicates that the codon is significantly underrepresented (RSCU value < 0.6), orange highlight indicates that the codon is significantly overrepresented (RSCU value > 1.6). Bold indicates the most preferred codon for each amino acid in each clade.

| **Amino acid** | **Codon** | **Asian SEA2b (CFB)** | **Cosmo AF1b (dog)** | **Asian SEA2a (dog)** | **Arctic A (Arctic fox)** | **Cosmo AM2a (mongoose)** | **RAC-SK SCSK (skunk)** | **Bat LC (hoary bat)** | **Bat EF-E2 (big brown bat)** | **Bat TB1 (Mexican free tailed bat)** | **Bat DR (vampire bat)** |
| --- | --- | --- | --- | --- | --- | --- | --- | --- | --- | --- | --- |
| Phe | UUU | 0.874 | 0.635 | 0.822 | 0.802 | 0.642 | **1.004** | 0.972 | **1.067** | **1.123** | 0.897 |
|  | UUC | **1.126** | **1.365** | **1.178** | **1.198** | **1.358** | 0.996 | **1.028** | 0.933 | 0.877 | **1.103** |
| Leu | UUA | 0.986 | 0.558 | 1.092 | 0.449 | 0.723 | 0.602 | 0.903 | 0.966 | 1.045 | 1.012 |
|  | UUG | **1.252** | 1.245 | 1.037 | 1.679 | **1.505** | **1.728** | **2.114** | **1.798** | **1.637** | **1.642** |
|  | CUU | 0.889 | 0.356 | 1.000 | 0.603 | 0.332 | 0.940 | 0.859 | 0.604 | 0.868 | 0.691 |
|  | CUC | 0.718 | 0.542 | 0.513 | 0.521 | 0.704 | 0.678 | 0.528 | 0.708 | 0.520 | 0.661 |
|  | CUA | 1.074 | **1.697** | 1.044 | 0.944 | 1.290 | 0.735 | 0.704 | 0.522 | 0.503 | 0.584 |
|  | CUG | 1.080 | 1.603 | **1.315** | **1.803** | 1.446 | 1.318 | 0.891 | 1.401 | 1.427 | 1.411 |
| Ile | AUU | **1.145** | 0.977 | **1.112** | **1.07** | **1.069** | 0.921 | 1.033 | 0.854 | 1.039 | **1.100** |
|  | AUC | 0.735 | 0.927 | 0.846 | 0.929 | 0.915 | 0.862 | 0.858 | 1.033 | 1.039 | 0.855 |
|  | AUA | 1.120 | **1.096** | 1.042 | 1.001 | 1.016 | **1.217** | **1.109** | **1.113** | 0.920 | 1.045 |
| Val | GUU | 1.179 | 0.997 | 1.018 | 1.089 | 0.989 | 1.011 | **1.280** | 1.068 | **1.224** | **1.192** |
|  | GUC | **1.333** | **1.454** | **1.381** | **1.139** | **1.458** | 1.073 | 1.004 | 0.738 | 0.932 | 0.954 |
|  | GUA | 0.655 | 0.563 | 0.943 | 0.881 | 0.548 | 0.622 | 0.711 | 0.749 | 0.634 | 0.728 |
|  | GUG | 0.833 | 0.985 | 0.658 | 0.892 | 1.005 | **1.295** | 1.004 | **1.444** | 1.210 | 1.126 |
| Ser | UCU | 1.222 | 1.286 | **1.557** | 1.222 | 1.181 | 1.276 | 1.407 | 1.699 | **1.729** | **1.579** |
|  | UCC | 1.232 | 0.793 | 1.000 | 0.842 | 0.895 | **1.535** | 0.640 | 0.702 | 0.783 | 0.776 |
|  | UCA | **1.604** | **1.846** | 1.272 | **1.733** | **1.886** | 1.274 | **1.741** | **1.818** | 1.581 | 1.535 |
|  | UCG | 0.486 | 0.735 | 0.588 | 0.658 | 0.667 | 0.607 | 0.305 | 0.389 | 0.320 | 0.445 |
|  | AGU | 0.973 | 0.981 | 1.057 | 0.842 | 1.048 | 0.645 | 1.269 | 0.895 | 0.955 | 1.019 |
|  | AGC | 0.481 | 0.359 | 0.527 | 0.703 | 0.324 | 0.663 | 0.640 | 0.497 | 0.631 | 0.646 |
| Pro | CCU | **1.241** | **1.560** | 1.047 | **1.652** | 1.417 | 1.020 | **1.632** | **1.615** | **1.766** | **1.939** |
|  | CCC | 1.000 | 0.928 | **1.202** | 1.172 | 1.083 | 0.856 | 0.735 | 0.721 | 0.984 | 0.601 |
|  | CCA | 1.017 | 0.891 | 0.945 | 1.168 | **1.472** | 0.936 | 0.941 | 0.539 | 0.492 | 0.513 |
|  | CCG | 0.741 | 0.620 | 0.806 | 0.007 | 0.028 | **1.188** | 0.691 | 1.125 | 0.758 | 0.948 |
| Thr | ACU | **1.398** | 1.141 | **1.509** | **1.539** | 1.353 | 1.358 | 0.978 | 0.964 | 1.417 | 1.307 |
|  | ACC | 0.954 | 1.243 | 0.879 | 0.721 | 0.837 | 0.981 | 1.368 | 1.353 | 0.777 | 0.838 |
|  | ACA | 1.198 | **1.463** | 1.026 | 0.690 | **1.489** | **1.460** | **1.515** | **1.415** | **1.546** | **1.622** |
|  | ACG | 0.450 | 0.153 | 0.586 | 1.051 | 0.320 | 0.199 | 0.139 | 0.268 | 0.258 | 0.233 |
| Ala | GCU | 1.322 | 1.000 | **1.507** | 1.348 | 1.058 | 1.482 | 0.852 | 1.129 | **1.484** | 1.389 |
|  | GCC | 1.027 | 1.078 | 0.794 | 0.879 | 0.990 | 0.725 | 0.982 | 0.990 | 0.883 | 0.805 |
|  | GCA | **1.503** | **1.710** | 1.494 | **1.634** | **1.695** | **1.705** | **1.804** | **1.836** | 1.153 | **1.413** |
|  | GCG | 0.148 | 0.211 | 0.205 | 0.139 | 0.257 | 0.088 | 0.363 | 0.046 | 0.480 | 0.393 |
| Tyr | UAU | **1.074** | **1.249** | **1.144** | **1.505** | **1.280** | **1.266** | 0.851 | **1.408** | **1.330** | **1.198** |
|  | UAC | 0.926 | 0.751 | 0.856 | 0.495 | 0.720 | 0.734 | **1.149** | 0.592 | 0.670 | 0.802 |
| His | CAU | 0.531 | **1.219** | 0.780 | 0.923 | **1.265** | **1.083** | **1.231** | **1.229** | **1.308** | **1.311** |
|  | CAC | **1.469** | 0.781 | **1.220** | **1.077** | 0.735 | 0.917 | 0.769 | 0.771 | 0.692 | 0.689 |
| Gln | CAA | **1.193** | **1.023** | **1.569** | **1.200** | 1.000 | 0.674 | **1.013** | **1.058** | **1.119** | 0.830 |
|  | CAG | 0.807 | 0.977 | 0.431 | 0.800 | 1.000 | **1.326** | 0.988 | 0.942 | 0.881 | **1.170** |
| Asn | AAU | 0.945 | **1.069** | 0.970 | **1.172** | 0.883 | **1.088** | 0.918 | **1.327** | **1.236** | **1.274** |
|  | AAC | **1.055** | 0.931 | **1.030** | 0.828 | **1.117** | 0.912 | **1.083** | 0.673 | 0.764 | 0.726 |
| Lys | AAA | **1.051** | 0.910 | 0.821 | 0.495 | 0.885 | 0.683 | 0.589 | 0.679 | 0.960 | 0.960 |
|  | AAG | 0.949 | **1.090** | **1.179** | **1.505** | **1.115** | **1.317** | **1.411** | **1.321** | **1.040** | **1.040** |
| Asp | GAU | **1.017** | 0.996 | 0.717 | **1.041** | 0.992 | **1.010** | 0.867 | 0.902 | 0.956 | 0.952 |
|  | GAC | 0.983 | **1.004** | **1.283** | 0.959 | **1.008** | 0.990 | **1.133** | **1.098** | **1.044** | **1.048** |
| Glu | GAA | 0.913 | 0.873 | 0.804 | 0.690 | 0.805 | **1.222** | 0.833 | 0.766 | 0.919 | 0.869 |
|  | GAG | **1.087** | **1.127** | **1.196** | **1.310** | **1.195** | 0.778 | **1.168** | **1.234** | **1.081** | **1.131** |
| Cys | UGU | **1.023** | **1.948** | 0.498 | **1.333** | **1.429** | **1.111** | **1.062** | **1.015** | **1.333** | **1.030** |
|  | UGC | 0.977 | 0.052 | 1.502 | 0.667 | 0.571 | 0.889 | 0.938 | 0.985 | 0.667 | 0.970 |
| Arg | CGU | 0.780 | 0.804 | 0.780 | 0.791 | 0.746 | 0.492 | 0.480 | 0.531 | 0.504 | 0.380 |
|  | CGC | 0 | 0.017 | 0 | 0 | 0.030 | 0.552 | 0.240 | 0.224 | 0.252 | 0.370 |
|  | CGA | 0.780 | 0.342 | 0.776 | 0.261 | 1.061 | 0.376 | 0.480 | 0.030 | 0 | 0.273 |
|  | CGG | 0.260 | 0.474 | 0.266 | 0.772 | 0.317 | 0.523 | 0.495 | 0.792 | 0.512 | 0.417 |
|  | AGA | **2.319** | **3.189** | **2.805** | **3.317** | **2.844** | **2.462** | **2.415** | **3.617** | **2.757** | **2.804** |
|  | AGG | 1.863 | 1.174 | 1.374 | 0.859 | 1.002 | 1.595 | 1.890 | 0.807 | 1.974 | 1.757 |
| Gly | GGU | 1.079 | 0.770 | 0.872 | 0.811 | 0.838 | 0.882 | 0.699 | 0.940 | 0.556 | 0.435 |
|  | GGC | 0.401 | 0.702 | 0.549 | 0.417 | 0.603 | 0.794 | 0.949 | 0.529 | 0.819 | 0.931 |
|  | GGA | 1.134 | 1.102 | 1.215 | 1.013 | 1.382 | **1.168** | 0.975 | 1.199 | **1.660** | **1.648** |
|  | GGG | **1.387** | **1.426** | **1.365** | **1.758** | **1.176** | 1.156 | **1.379** | **1.332** | 0.966 | 0.986 |

**Supplementary Table S3: PCA loadings for each codon on PC1 and PC2.**

| **Amino acid** | **Codon** | **PC1** | **PC2** | **PC3** |
| --- | --- | --- | --- | --- |
| F | TTT | -0.1238798 | 0.14969785 | -0.1538584 |
|  | TTC | 0.12312555 | -0.1532243 | 0.15259887 |
| L | TTA | -0.0812072 | 0.22367578 | 0.02686364 |
|  | TTG | -0.1129219 | -0.0225038 | -0.1916261 |
|  | CTT | 0.02306152 | 0.26314298 | -0.0511886 |
|  | CTC | -0.1000353 | 0.04885798 | -0.0145167 |
|  | CTA | 0.1135647 | -0.1556209 | 0.20204684 |
|  | CTG | 0.07898578 | -0.1565916 | -0.1300544 |
| I | ATT | 0.08630567 | 0.06288623 | 0.01381255 |
|  | ATC | -0.0620328 | -0.1352308 | -0.1030095 |
|  | ATA | -0.0041873 | -0.1197346 | 0.12262433 |
| V | GTT | -0.0542682 | 0.09606773 | -0.0303476 |
|  | GTC | 0.15332263 | -0.0518635 | 0.22602061 |
|  | GTA | 0.1380224 | 0.1369166 | -0.1354166 |
|  | GTG | -0.1946201 | -0.0896962 | -0.07509 |
| S | TCT | -0.1495211 | 0.15423219 | -0.0620752 |
|  | TCC | 0.08025414 | 0.14053837 | 0.07468375 |
|  | TCA | -0.0820463 | -0.1959373 | -0.000233 |
|  | TCG | 0.13319661 | -0.1151713 | 0.09597072 |
|  | AGT | -0.0534769 | 0.12409925 | 0.12714272 |
|  | AGC | -0.0260652 | 0.1144503 | -0.1953197 |
| P | CCT | -0.1396412 | -0.1177935 | -0.1435618 |
|  | CCC | 0.20641153 | 0.02744473 | -0.0499682 |
|  | CCA | 0.19515143 | -0.045743 | 0.01343401 |
|  | CCG | -0.1603017 | 0.09819678 | 0.06693523 |
| T | ACT | 0.10407946 | 0.11476606 | -0.14015 |
|  | ACC | -0.0981148 | -0.1134341 | 0.10882896 |
|  | ACA | -0.2371434 | -0.0057607 | 0.120301 |
|  | ACG | 0.18184363 | 0.02786802 | -0.2112205 |
| A | GCT | 0.05003959 | 0.18662366 | -0.1271572 |
|  | GCC | -0.0089584 | -0.1729656 | 0.12979821 |
|  | GCA | 0.05665423 | -0.1914721 | 0.04631538 |
|  | GCG | -0.1365341 | 0.06016717 | 0.04883212 |
| Y | TAT | 0.0406744 | -0.132285 | -0.2318439 |
|  | TAC | -0.041297 | 0.13122295 | 0.23190373 |
| H | CAT | -0.2084364 | -0.1216455 | -0.0120494 |
|  | CAC | 0.20005047 | 0.14040413 | 0.00407316 |
| Q | CAA | 0.20443162 | 0.13159382 | 0.00098762 |
|  | CAG | -0.1674602 | -0.1455563 | 0.03870188 |
| N | AAT | -0.1227503 | -0.0262692 | -0.2027536 |
|  | AAC | 0.14256955 | 0.0729617 | 0.19341221 |
| K | AAA | -0.0862157 | 0.03246694 | 0.23386082 |
|  | AAG | 0.10029931 | -0.0907991 | -0.204241 |
| D | GAT | -0.0507114 | -0.1465419 | 0.00189217 |
|  | GAC | 0.02860627 | 0.19559701 | 0.05931421 |
| E | GAA | -0.1028884 | 0.00177118 | 0.17134389 |
|  | GAG | 0.14081741 | -0.0689321 | -0.1470548 |
| C | TGT | -0.0306328 | -0.2471806 | 0.11614228 |
|  | TGC | 0.03588002 | 0.24888704 | -0.0826329 |
| R | CGT | 0.22584217 | -0.0709505 | 0.07567385 |
|  | CGC | -0.2191445 | 0.06700388 | -0.0692887 |
|  | CGA | 0.14547106 | 0.12879737 | 0.17176766 |
|  | CGG | -0.0233685 | -0.1253969 | -0.2416757 |
|  | AGA | 0.00143952 | -0.1466299 | -0.1945023 |
|  | AGG | -0.1182627 | 0.17147498 | 0.10522635 |
| G | GGT | 0.15645291 | -0.0134029 | 0.00694933 |
|  | GGC | -0.1959211 | -0.0051093 | 0.1054936 |
|  | GGA | -0.1587444 | 0.09814174 | -0.0294782 |
|  | GGG | 0.20129191 | -0.1132681 | -0.0777299 |


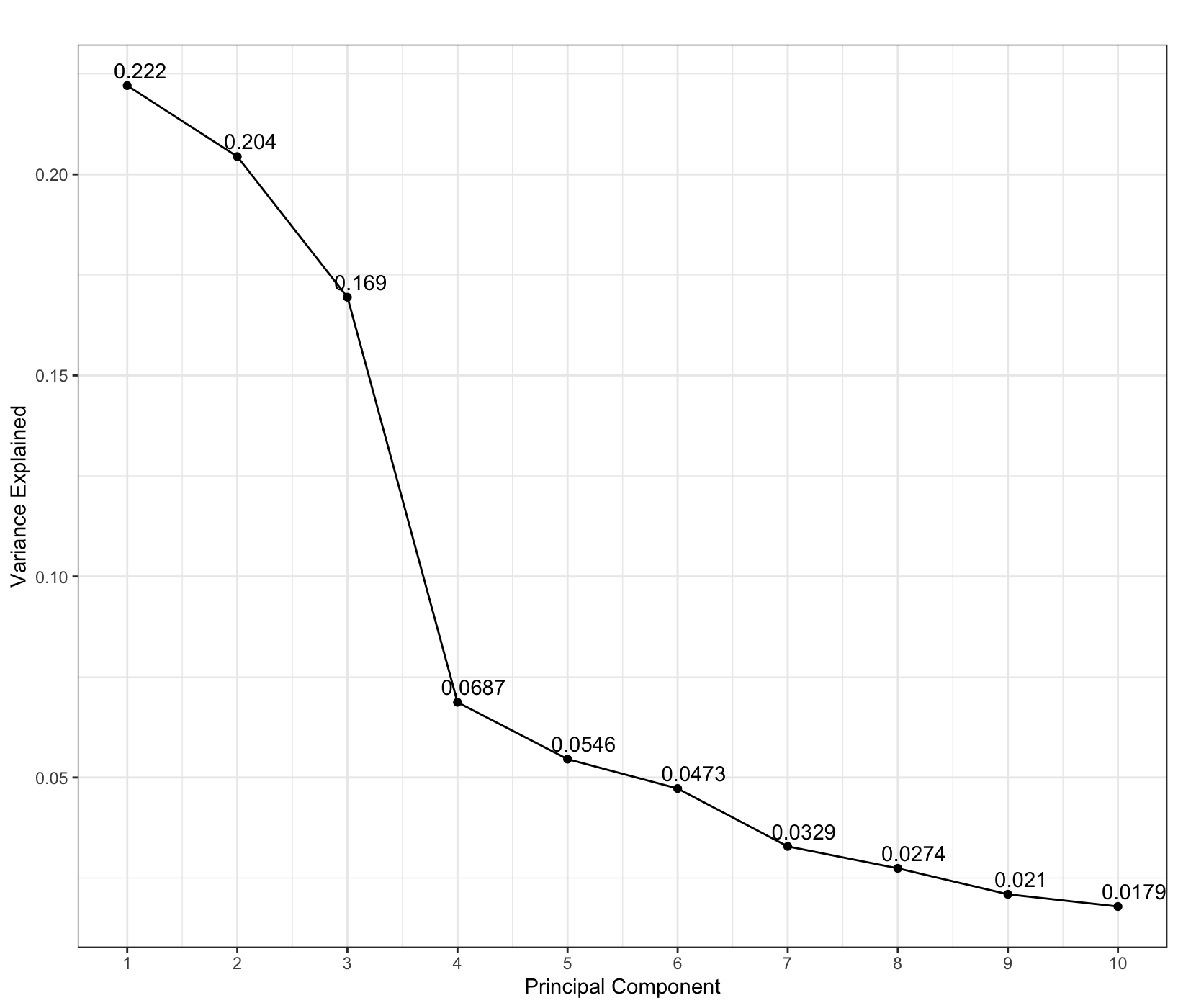


**Supplementary Figure S1: Scree plot for the PCA of raw codon usage values**.


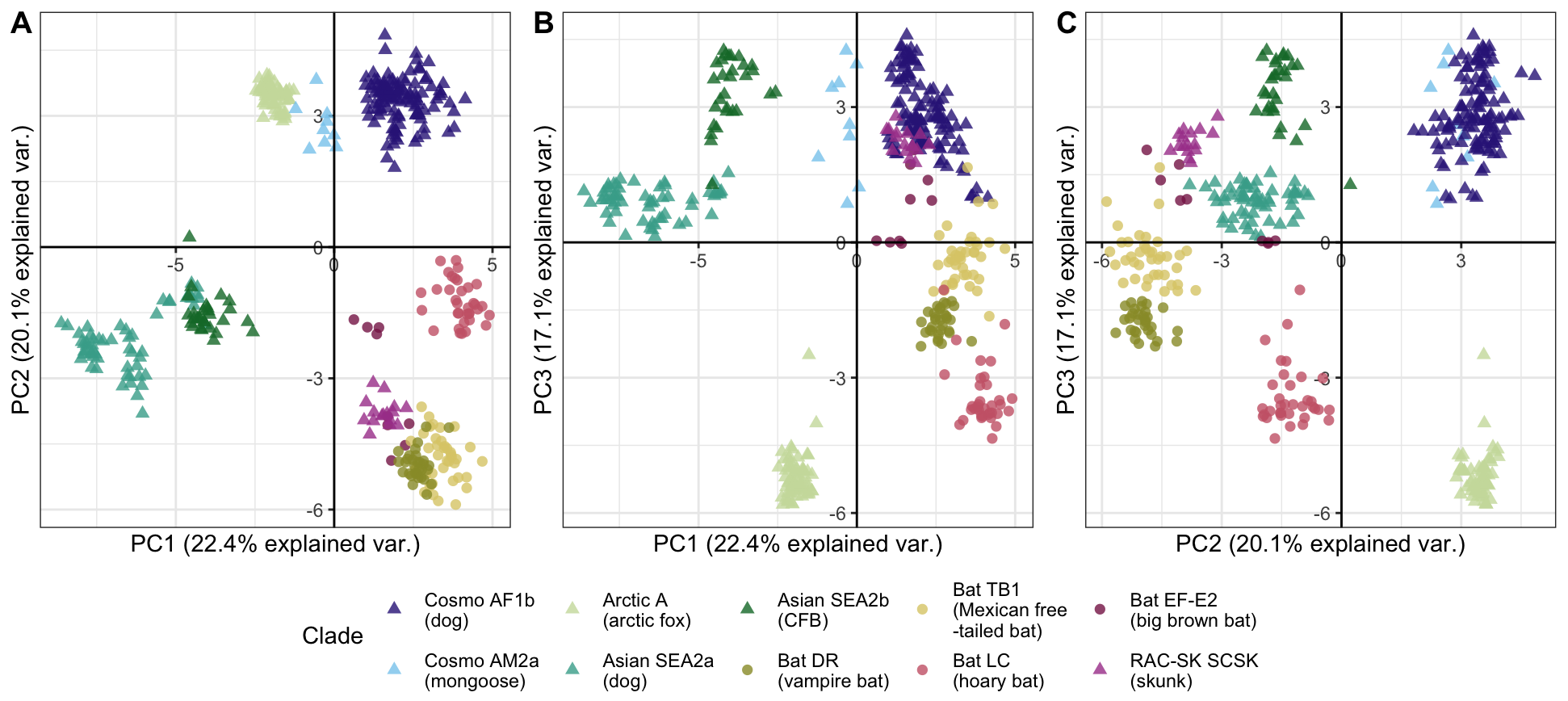


**Supplementary Figure 2: PCA using RSCU values.**
